# Antimicrobial resistance and molecular characterization of *Klebsiella* species causing bovine mastitis in Nghe An province, Vietnam

**DOI:** 10.5455/javar.2023.j662

**Published:** 2023-03-31

**Authors:** My Trung Tran, Duc Minh Vu, Manh Duy Vu, My Thi Phuong Bui, Binh Xuan Dang, Lan Thi Mai Dang, Thien Van Le

**Affiliations:** 1 Thai Nguyen University of Agriculture and Forestry, Thai Nguyen University, Thai Nguyen, Vietnam; 2 College of Economics and Technology, Thai Nguyen University, Thai Nguyen, Vietnam; 3 TH Milk Food Joint Stock Company, Nghia Son, Nghia Dan, Vietnam; 4 Vietnam National University of Agriculture, Ha Noi, Vietnam

**Keywords:** Antimicrobial resistance, Klebsiella spp., mastitis, multidrug-resistant, virulence factors

## Abstract

**Objectives:**

This study aimed to determine the antibiotic-resistant profile and to identify molecular characterization of some virulence genes of *Klebsiella *spp. isolated from mastitis samples in Vietnam.

**Materials and Method:**

A total of 468 samples from clinical mastitis cases were collected and submitted to the Laboratory. All samples were cultured, and *Klebsiella *spp. was identified through biochemical reactions and confirmed by Polymerase chain reaction (PCR). Antimicrobial resistance was tested by disk diffusion method, and virulence and resistance genes were tested by PCR.

**Results:**

An antibiogram study showed that a high proportion of isolates are multidrug-resistant (94%). All isolates were resistant to lincomycin and sulfamethoxazole, followed by ampicillin (94%), sulphonamide (66%), amoxicillin (56%), streptomycin (52%), polymyxin B (28%), colistin sulfate (12%), tetracycline (6%), ciprofloxacin (4%), florfenicol (4%), enrofloxacin (4%), piperacillin (2%), trimethoprim (2%), nalidixic acid (2%), imipenem (2%), and sulfamethoxazole/trimethoprim (2%). In contrast, all isolates showed sensitivity to gentamicin and ceftiofur. The appearance of an efflux pump system, extended-spectrum beta-lactamase (ESBL), tetracycline, and sulphonamides-resistant genes was reconfirmed using different specific primers. Capsular serotype K1 and virulence genes *mag*A, *fim*H, and *ent*B, responsible for hypermucoviscosity production, adherence, and enterobactin production, were confirmed in isolates. Multidrug resistance and virulence potential in *Klebsiella *spp. are changing this mastitis pathogen into a superbug and making its management harder.

**Conclusions:**

*Klebsiella *spp. associated with bovine mastitis in Nghe An province were mostly multidrug-resistant and carried virulence genes including *fim*H, *ent*B, and antimicrobials resistant genes (*bla*_SHV_, *acr*A_Kp_, *tet*A, etc.), but these isolates were not ESBL producers.

## Introduction

Mastitis is a considerable and recurring economic loss in the dairy industry worldwide. It differs from other animal diseases because several kinds of bacteria can cause the disease; these pathogens colonize the udder, multiply, and produce harmful substances that result in inflammation, decreased milk production, and affected milk quality [1]. Mastitis is an important and economically significant disease in the dairy industry, and not less so in countries like Vietnam, where the dairy sector is still in a relatively early phase of development. Economic effects of this disease include decreased milk production from affected animals, changes in milk quality, discarded milk, veterinary costs, and replacement costs associated with the culling of chronically affected cows and mortality [2]. The average failure costs of mastitis per cow per year were $US131 in the US [3] and 662 Canadian dollar in Canada [1]; milk production dropped accounts for approximately 70% of the total cost of mastitis [4]. In a previous study, we reported that the prevalence of bovine mastitis in Vietnam was 2.9%, and the isolation result of *Klebsiella* was 21.0% (unpublished data).

*Klebsiella *is a genus of Gram-negative, oxidase-negative, rod-shaped bacteria with a prominent polysaccharide-based capsule [5]. *Klebsiella* species are found everywhere in nature. This pathogen can be found in water, soil, plants, insects, and other animals, including humans [6]. Clinical mastitis due to *Klebsiella* is often severe and does not respond well to antimicrobial treatment, resulting in a prolonged duration of intramammary infection and substantial loss of milk yield [7]. Many virulence factors may cause udder infection, and its severity includes capsular serotypes, iron-scavenging systems, and fimbriae have been reported to be involved in the virulence of *Klebsiella pneumoniae* of human origin [8]. Recently, a study about hypermucoviscous phenotype related to the adhesive and invasive ability of the bovine mammary epithelial cells [9], and potential virulence genes isolated from clinical mastitis on large dairy farms have been reported in China [10]. 

In Vietnam, there are few studies only on the prevalence of mastitis but not on any specific pathogens and their virulence factors. Therefore, studying the virulence factors of *Klebsiella* spp. isolated in Vietnam’s large dairy farms is important. The objectives of this study were (1) to determine the antibiotic-resistant profile and (2) to identify molecular characterization of some virulence genes of *Klebsiella *spp. isolated. The results of this study would be useful for farmers or veterinarians to consider and/or make decisions to minimize the loss during and after episodes of mastitis caused by *Klebsiella *spp.

## Materials and Methods

### Herds profile and samples

This study was conducted from several large dairy farms in Nghe An province, Vietnam, in 2022. The animal population consisted of Holstein-Friesian dairy cows from intensively managed, fully housed, and total mixed ration-fed, ranging in average size from 1.800 to 4.500 animals. A total of 468 samples were collected [under aseptic conditions (sterilized sample vials, clean udder(s), sampling, labeling, refrigeration post-collection, and transport to the lab in the icebox)], submitted, and cultured on the day of arrival.

### Bacteria isolation and identification 

A loopful of mastitis sample (10 μl) was the first culture onto blood agar (incubated at 37°C for 24–48 h); the sample was considered culture-positive if 1 or more colonies were observed (≥100 CFU/ml) [11], Gram-negative and rod shape checked by Gram stain and microscope then inoculated on MacConkey agar and Chromatic agar (Liofilchem) (incubate at 37°C for 24 h). A mucoid, light pink colony on MacConkey agar and a green-blue colony on Chromatic agar were selected for confirmation by biochemical tests. Biochemical tests for *Klebsiella* include indole production, citrate utilization, motility [12], other carbohydrate fermentation (glucose, sucrose, lactose), gas production, methyl red—Voges Proskauer, oxidase, and catalase also tested.

### Antimicrobial susceptibility test

Antimicrobial susceptibilities were conducted by using the disc diffusion test (DDT) with the Mueller-Hinton agar (MHA) technique which is described in Clinical and Laboratory Standards Institute (CLSI) M100-30th [13]. The bacteria were spread over the surface of sterile MHA plates using sterile cotton swabs then antibiotics discs were placed over the surface of inoculated plates. Let’s plate dry on a vertical surface for 15 min and then incubate at 37°C for 16–24 h. The zone of inhibitions of each antibiotic was recorded in millimeters (mm), corresponding to the CLSI standard values of respective antibiotics. Multi-drug resistance (MDR) was defined as acquired non-susceptibility to at least one agent in three or more antimicrobial categories [14]. Antimicrobial agents used in this study were listed and described in Table 1.

### Extended-spectrum beta-lactamase (ESBL) production

ESBL production tests followed CLSI M100-30th [13] using the disk-diffusion method with MHA. Two sets of antibiotics were ceftazidime 30 μg together with ceftazidime-clavulanate 30/10 μg and cefotaxime 30 μg together with cefotaxime-clavulanate 30/10 μg. The inoculum was prepared according to the standard disk diffusion procedure. The agar plate was then incubated at 35°C ± 2°C for 16–20 h. The result was interpreted as ESBL positive when the inhibition zone of either antimicrobial agent tested in combination with clavulanate was bigger than the zone diameter of the agent when tested alone over 5 mm, as shown in Table 2.

### Deoxyribonucleic acid (DNA) extraction

DNA of each isolate was extracted using Chelex 100 Resin protocol [15]: colonies on the agar were collected and enriched in peptone water overnight, and the medium (1.5 ml) was then centrifuged in 800 × g, for 5 min at 4°C. Supernatant collected (500 μl) and then centrifuged at 13,000 × g, 5 min at 4°C, remove supernatant, then add 200 μl Chelex 100 (in TE buffer) and mix well. Incubate with shaking at 56°C, 30 min at 800 rpm, then vortex the vial for 10 sec and incubate again at 96°C, 8 min at 800 rpm. The vial was then mixed and centrifuged at 13,000 × g, 3 min at 4°C, taking 100 μl supernatant for PCR running. DNA extracted was stored in the Eppendorf tube and kept under −20°C until the PCR reaction was carried out.

**Table 1.** Antimicrobials used for this study.

Antimicrobial	Abbreviation	Code	Concentration
Ceftiofur	EFT	EFT	30 μg
Gentamicin	GEN	CN120	120 μg
Sulfamethoxazole/Trimethoprim	STX	SXT25	1.25/23.75 μg
Imipenem	IMI	IPM10	10 μg
Nalidixic acid	NAL	NA30	30 μg
Trimethoprim	TRI	W5	5 μg
Piperacillin	PIP	PRL100	100 μg
Enrofloxacin	ENR	ENR5	5 μg
Florfenicol	FLO	FFC30	30 μg
Ciprofloxacin	CIP	CIP5	5 μg
Tetracycline	TET	TE30	30 μg
Colistin sulfate	COL	CT10	10 μg
Polymyxin B	POL	PB300	300 ui
Streptomycin	STR	S10	10 μg
Amoxycillin	AMO	AML10	10 μg
Sulphonamide	SUL	S3 300	300 μg
Ampicillin	AMP	AMP10	10 μg
Sulfamethoxazole	SUX	RL25	25 μg
Lincomycin	LIN	MY15	15 μg

**Table 2.** Criteria to confirm ESBL production.

Single antibiotic diameter zone	Combination diameter zone
Ceftazidime < 22 mm	Ceftazidime-clavulanate ^≥^ 27 mm
Cefotaxime < 27 mm	Cefotaxime-clavulanate ^≥^ 32 mm

### Molecular confirmation of isolates

The *Klebsiella* isolates were first identified by biochemical reactions and then confirmed by using *Klebsiella-*specific gene *gyrA* (441 bp) [16]; the test followed the standard protocol [15]: Polymerase chain reaction (PCR) (20 μl) was prepared by mixing 10 μl master mix (MyTaq HS Red Mix), with 1 μl of each forward (F-CGC GTA CTA TAC GCC ATG AAC GTA) and reverse (R-ACC GTT GAT CAC TTC GGT CAG G) primers, 3 μl nuclease-free PCR water, and 5 μl DNA sample, 100–1,500 bp DNA ladder (PCRBIO Ladders IV) was used. The mixture was first run at 95°C for denaturing for 3 min and 35 cycles of 95°C for 15 sec, 62°C for 15 sec, and 72°C for 10 sec on a thermal cycler (Biometra Professional, Germany). DNA extracted from *Klebsiella oxytoca* American Type Culture Collection (ATCC) 49131 and *Staphylococcus aureus* ATCC 25923 were used as the positive and negative control, respectively. PCR products were then purified and identified by agar electrophoresis (Mupid-Exu) and transillumination (Major Science - MBE 200A).

### Confirmation of drug-resistance genes in isolates

*Klebsiella *isolates were confirmed again some specific genes corresponding resistance to ESBL production by *bla*_SHV_, *bla*_TEM_, *bla*_KPC_, *bla*_NDM_, *bla*_CTX-M-3_ and *bla*_IMP_, to Sulphonamides by *sul1, sul2*, to Tetracycline by *tet*A*, tet*B, to Trimethoprim by *DHFR-I* and to Quinolone by *qnr*A. The reaction mixture was prepared, and the tests followed the protocol described earlier with primers, sequences, predicted sizes, and annealing temperatures described in Table 3. 

### Detection of virulence factors

The virulence factors associated with *Klebsiella *spp. were determined by targeting specific gene *fimH* encoding for type 1 fimbriae, *rmpA* gene for the regulator of the mucoid phenotype A, *magA* for hypermucoviscosity production (mucoviscosity-associated gene A), *K1 *&* K2* for encoding capsule serotype, *iro*N, *ent*B, and* iut*A genes for encoding siderophore. The reaction mixture was prepared, and the tests followed the earlier protocol with primers, sequences, predicted sizes, and annealing temperatures described in Table 4. 

**Table 3.** The primer sequence, target gene, and amplicon size of antibiotics resistance genes used in this study.

Gene	Primer sequences (5’–3’)		Annealing temp	Size (bp)	Reference
	β-lactamases				
*bla*_SHV_	F	CTTTATCGGCCCTCACTCAA	53	237	[17]
	R	AGGTGCTCATCATGGGAAAG			
*bla*_TEM_	F	CGCCGCATACACTATTCTCAGAATGA	59	444	[17]
	R	ACGCTCACCGGCTCCAGATTTAT			
*bla*_KPC_	F	CGTCTAGTTCTGCTGTCTTG	56	798	[18]
	R	CTTGTCATCCTTGTTAGGCG			
*bla*_NDM_	F	GGTTTGGCGATCTGGTTTTC	56	621	[18]
	R	CGGAATGGCTCATCACGATC			
*bla*_CTX-M-3_	F	AATCACTGCGTCAGTTCAC	53	701	[19]
	R	TTTATCCCCCACAACCCAG			
*bla*_IMP_	F	GGAATAGAGTGGCTTAAYTCTC	56	232	[20]
	R	GGTTTAAYAAAACAACCACC			
Sulfonamides					
*sul*1	F	TTCGGCATTCTGAATCTCAC	50	822	[21]
	R	ATGATCTAACCCTCGGTCTC			
*sul*2	F	CGGCATCGTCAACATAACC	54	722	[19]
	R	GTGTGCGGATGAAGTCAG			
Tetracycline					
*tet*A	F	GGTTCACTCGAACGACGTCA	57	577	[22]
	R	CTGTCCGACAAGTTGCATGA			
*tet*B	F	CCTCAGCTTCTCAACGCGTG	56	634	[21]
	R	GCACCTTGCTGATGACTCTT			
Trimethoprim					
*DHFR-I*	F	AAGAATGGAGTTATCGGGAATG	56	391	[19]
	R	GGGTAAAAACTGGCCTAAAATTG			
Quinolone					
*qnr*A	F	ATTTCTCACGCCAGGATTTG	54	516	[23]
	R	GATCGGCAAAGGTTAGGTCA			
Multidrug efflux pump					
*acr*A_Kp_	F	ATTTCTCACGCCAGGATTTG	55	940	[24]
	R	GATCGGCAAAGGTTAGGTCA			

### Statistics analysis

Spreadsheets were used as software tools for data entry, storage, analysis, and visualization in this study.

## Results

### The distribution of Klebsiella spp. in bovine mastitis in Nghe An province, Vietnam

**Figure 1.**
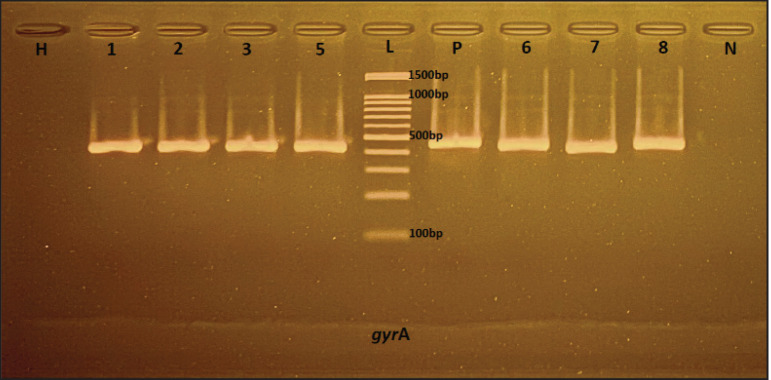
PCR reaction assay result for *Klebsiella* identification; ladder (L); positive control *gyr*A (441 bp) (P); positive samples (1–5, 6–8); negative control (N).

From 468 samples submitted and tested during the study period, we followed the isolation protocols of the Laboratory and identified 103 *Klebsiella *strains by biochemical reactions. Because we did not investigate the prevalence and the sources of this pathogen in the farm (e.g., animals, water, bedding materials, milking equipment, etc.) but were interested in the virulence genes and antimicrobial resistance and its related genes. Thus, 50 isolates were randomly selected from 103 strains for further study and were confirmed as *Klebsiella *spp. by the support of PCR through the *gyrA* gene (Fig. 1). All isolates came from clinical mastitis cases from dairy farms in large-scale herds in Vietnam. 

### Antimicrobial resistance and ESBL producing of Klebsiella spp. isolated

**Figure 2.**
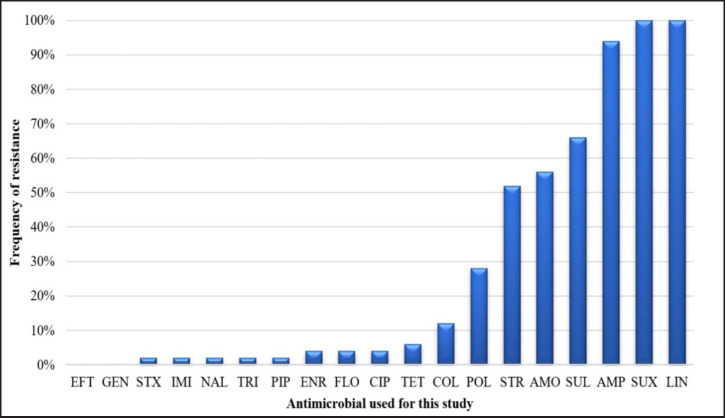
Drug resistance proportion of *Klebsiella* spp. isolates against antimicrobials used. Of 19 antibiotics used, we analyzed and demonstrated the results of MDR in Table 5.

All *gyrA* gene positives were then processed for antibiogram studies again, 19 common antibiotics (950 antibiograms) belong to 9 classes, and resistance levels varied between antibiotics (Fig. 2). All isolates were fully resistant to LIN and SUX, followed by AMP at 94% and SUL at 66%. Resistant to AMO, STR, and POL was 56%, 52%, and 28%, respectively. Resistance against Colistin (COL) was 12%, while TET was 6%; CIP, FLO, and ENR were at 4% each drug, followed by PIP, TRI, NAL, IMI, and SXT at 2% each. None of the isolates were resistant to GEN and EFT. 

**Table 4.** The primer sequence, target gene, and amplicon size of virulence genes used in this study.

Gene		Primers sequences (5’–3’)	Annealing temp	Size (bp)	Reference
*fim*H	F	ATGAACGCCTGGTCCTTTGC	59	688	[25]
	R	GCTGAACGCCTATCCCCTGC			
*rmp*A	F	ACTGGGCTACCTCTGCTTCA	55	516	[26]
	R	CTTGCATGAGCCATCTTTCA			
*mag*A	F	GGTGCTCTTTACATCATTGC	53	1283	[26]
	R	GCAATGGCCATTTGCGTTAG			
*K1*	F	GTAGGTATTGCAAGCCATGC	52	1046	[26]
	R	GCCCAGGTTAATGAATCCGT			
*K2*	F	GACCCGATATTCATACTTGACAGAG	52	641	[26]
	R	CCTGAAGTAAAATCGTAAATAGATGGC			
*iro*N	F	AAGTCAAAGCAGGGGTTGCCCG	63	665	[20]
	R	GACGCCGACATTAAGACGCAG			
*ent*B	F	ATTTCCTCAACTTCTGGGGC	60	371	[27]
	R	AGCATCGGTGGCGGTGGTCA			
*iut*A	F	GGCTGGACATCATGGGAACTGG	61	302	[20]
	R	CGTCGGGAACGGGTAGAATCG			

One isolate was found to have no sensitivity to 10 different drugs, and none of these isolates were resistant to only 1 drug, most resistant to 5 (9/50) or 6 (14/50) drugs. The 50 isolates resistant to 2–10 different drugs were presented in 23 patterns (Table 5). All strains tested and three isolates give results of resistance to two antibiotic classes which take account of 6%, and MDR (resistance to ≥3 antimicrobial classes) was 94% (47/50) of the total isolates tested. ESBL producing was tested for all isolates; fortunately, all strains were confirmed, not ESBL producers. 

### Detection of some antimicrobial resistance genes

**Figure 3.**
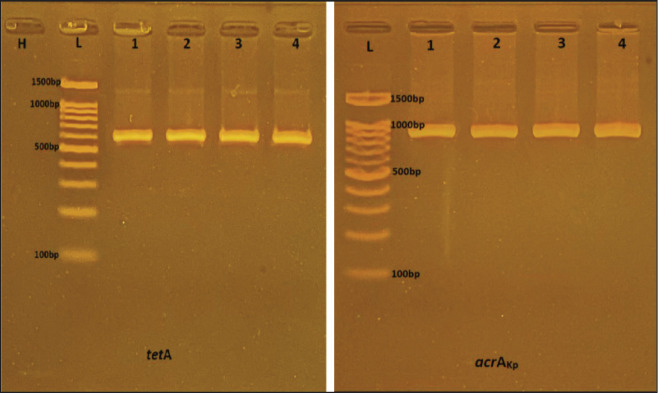
PCR result for *tet*A (1–4) 577 bp, *acr*A_Kp_ (5–8) 940 bp genes, H_2_O for negative control (H), ladder (L).

The results of some antimicrobial resistance genes are summarized in Table 6 and Figure 3. All strains were tested for 13 different genes representatives for 5 types of antimicrobials. We found that 100% of isolates carried gene *acr*A_Kp_, which might relate to the MDR found. Resistance towards Beta-lactam antibiotics was found with 94% of isolates carrying *bla*_SHV_, and 8% *bla*_NDM_, and no strains were found positive with *bla*_TEM_, *bla*_KPC_, *bla*_CTX-M-3_, and *bla*_IMP_. We found that the proportion of isolates that harbored *sul*1 and *sul*2 was 4% and 2%, respectively. Tetracycline-resistant encoded genes were found in 100% of isolates with *tet*A, but none carried *tet*B genes. *qnr*A gene encoding quinolone-resistant was not found in all *Klebsiella* spp. strains in this study. Analyzed gene patterns, we found 1 isolate with 5 genes (tetA-* bla*_SHV_ -* acr*A_Kp_ -*sul*1-*sul*2), 1 isolate with the pattern (*tet*A-* bla*_NDM_ -* acr*A_Kp_ -*sul*1), another isolate with (*tet*A-* bla*_SHV_ -* bla*_NDM_ -* acr*A_Kp_), 45 isolates with 3 genes (*tet*A-* bla*_SHV_ -* acr*A_Kp_) and 2 isolates with (*tet*A-* bla*_NDM_ -* acr*A_Kp_) patterns. 

**Table 5.** Antimicrobial resistance patterns of *Klebsiella *spp. isolates.

Resistance pattern	Number of drugs	Number of antimicrobial classes	Number of isolates
AMP-SUX-ENR-FLO-SUL-CIP-AMO-LIN-NAL-STR	10	6	1
AMP-SUX-FLO-TET-SUL-STX-LIN-TRI	8	5	1
AMP-SUX-SUL-AMO-LIN-COL-POL-PSUX	8	3	1
AMP-SUX-SUL-AMO-LIN-COL-POL-STR	8	4	2
AMP-SUX-SUL-AMO-LIN-POL-STR	7	4	4
AMP-SUX-TET-SUL-AMO-LIN-STR	7	5	2
AMP-SUX-SUL-AMO-LIN-COL-POL	7	3	1
AMP-SUX-ENR-CIP-AMO-LIN	6	4	1
AMP-SUX-SUL-AMO-LIN-STR	6	4	8
AMP-SUX-SUL-IMI-LIN-STR	6	5	1
AMP-SUX-AMO-LIN-COL-STR	6	4	1
AMP-SUX-SUL-AMO-LIN-POL	6	3	2
AMP-SUX-SUL-LIN-COL-POL	6	3	1
AMP-SUX-SUL-AMO-LIN	5	3	2
AMP-SUX-AMO-LIN-POL	5	3	1
AMP-SUX-SUL-LIN-STR	5	4	5
AMP-SUX-SUL-LIN-POL	5	3	1
AMP-SUX-AMO-LIN	4	3	2
AMP-SUX-LIN-STR	4	4	2
AMP-SUX-SUL-LIN	4	3	1
AMP-SUX-LIN-POL	4	3	1
AMP-SUX-LIN	3	3	6
SUX-LIN	2	2	3
Total			50

**Table 6.** Percentage and number of resistance genes detected among *Klebsiella* spp. isolates.

Virulence genes	Total isolates tested	Positive isolates	Positive proportion
*acrA*_Kp_	50	50	100%
*bla*_SHV_	50	47	94%
*bla*_TEM_	50	0	0%
*bla*_CTX-M-3_	50	0	0%
*bla*_KPC_	50	0	0%
*bla*_NDM_	50	4	8%
*bla*_IMP_	50	0	0%
*sul*1	50	2	4%
*sul*2	50	1	2%
*tet*A	50	50	100%
*tet*B	50	0	0%
DHFR-I	50	0	0%
*qn*rA	50	0	0%

### Detection of some virulence genes

**Figure 4.**
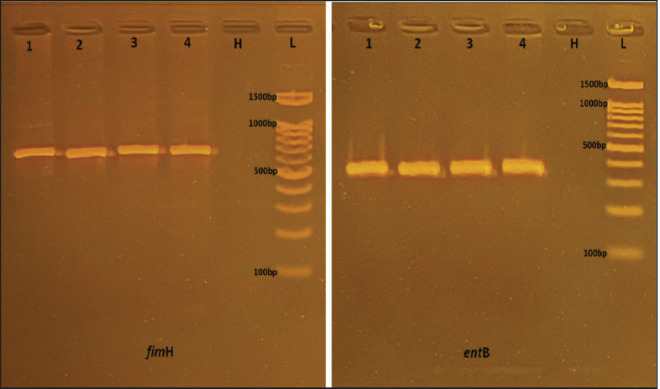
PCR result for *fim*H (1–4) 688 bp (left), *ent*B (1–4) 371 bp (right) genes, H_2_O for negative control (H), ladder (L).

Table 7 summarizes the results of virulence genes tested in this study. Fifty *Klebsiella* spp. isolates were investigated for some virulence genes; we found that 2 isolates harbored K1 (4%), all strains carried *fim*H (100%), and 49 *Klebsiella* spp. positive with *ent*B (98%) genes. None of these isolates harbored K2, *mag*A,* rmp*A, and *iut*A genes (Fig. 4).

**Table 7.** Percentage and number of virulence genes detected among *Klebsiella* spp. isolates.

Virulence genes	Total isolates tested	Positive isolates	Positive proportion
*K1*	50	2	4%
*K2*	50	0	0%
*magA*	50	0	0%
*rmpA*	50	0	0%
*fimH*	50	50	100%
*entB*	50	49	98%
*iutA*	50	0	0%

## Discussion

Our study revealed that *Klebsiella *spp. totally resistant to LIN and SUX, and AMP, resistant to LIN, was surprised as this antimicrobial has not been used by the farm, especially for treating mastitis cases; thus, a resistant gene related to this antimicrobial was not tested. Resistance to AMP was supported by similar studies in China [28] with 100% resistance, in Pakistan with 98% resistance to LIN, 86% AMP, and 99% SUX to mastitis pathogens [29]. Resistance toward AMP was used to predict the resistance to AMO [30]; thus, our AMO resistance was found to be high (56%) together with AMP, especially when compared with similar findings in China with 23.5% [31]. These drugs have been widely used in farms for a long time to deal with clinical mastitis, which may be why resistance to these antimicrobials is high. Fifty-two percent of isolates resistant to STR in the current study is higher in other countries like Canada with 38% [32], and China with 30.1% [28]. Like LIN, STR was never used by these farms before, but the resistance rate was still very high, even higher than in other countries close to Vietnam; it is potentially because this pathogen entered farms through bedding materials purchased and needs further studies to conclude. SUL was widely used in farms for treating mastitis, which might result from resistance to this drug up to 66%. This resistance was reported at 45.1% in China [33], which is quite lower than in our study. Resistance to TET was reported at 32% in China [34], 19% in Canada [32], and 41.5% in Brazil [6]. COL is a last resort antibiotic to treat infections caused by *K. pneumoniae*, and the resistance rate to this antibiotic has increased in recent years from 4.8% in 2013–2018 to 8.2% in 2019–2021 [35]; our resistance rate still higher, when compared to this finding and might come from the transmission between strains as this antimicrobial, was used for treatment calves with diarrhea. CIP, ENR, PIP, IMI, and STX were highly sensitive in our study, which could be a good choice for mastitis treatment, especially CIP, PIP, and IMI, as these drugs had never been used before at studied farms; this finding was supported by other studies in Brazil [6], in China [31]. We found that all isolates were sensitive to GEN and EFT; these drugs also be a good option for veterinarians to deal with mastitis cows. EFT was widely used in dairy farms as this drug generally results in a 0-h withdrawal time for the milk with a dose of 1 mg/kg body weight [36]. GEN was not commonly used for lactating animals and was banned in some countries, but not in Vietnam; thus, we recommended that farms consider this drug for treating diseases in calves only.

Our study revealed that 96% of isolates were MDR (Table 5). In same purpose studies in China [34,31,28] reported that 6.1% (4/66), 19.1%, and 43.9% *Klebsiella *spp. isolated from mastitis was MDR. Compared to these findings, our results are much higher, which indicates a quite high level and a possible association between antimicrobial resistance widespread among bacterial strains from animals and maybe from humans to animals and uncontrol antibiotic use.

The worldwide spread of *K. pneumoniae* producing extended-spectrum β-lactamase (ESBL-Kp) is a significant problem in human and veterinary medicine. Fortunately, all isolates in our study were not ESBL producers, similar to a report from Brazil 98.2% ESBL negative [6] and 98.8% in France [37]. This result indicated that *Klebsiella *spp. from dairy mastitis might not often be ESBL producers, and attention should be paid to β-lactams as it is an option for mastitis treatment.

It was revealed that there is a plasmid that carries a part of the *acr*AKp gene coding for a multidrug efflux pump (AcrAb) belonging to the resistance-nodulation-cell-division family [24]. Our results revealed that 100% of isolates carried gene *acr*AKp, which might relate to the MDR found. A study on the role of this gene revealed that AcrAB was involved in resistance to quinolones, other antimicrobial agents, and host antimicrobial peptides. 

The development of resistance to β-lactam antibiotics is attributed to the production of β-lactamases coded by different genes, including *bla*_SHV_, *bla*_TEM_, *bla*_NDM_, *bla*_CTX-M-3_, [38]. The prevalence of *bla*_SHV_ gene might explain the resistance to AMP and AMO in our study, as this gene often relates to the ability to hydrolyze penicillin and cephalosporins. A study in Iran reported that the proportion of *bla*_CTX_, *bla*_SHV_, and *bla*_TEM_ genes were 62.5%, 42.5%, and 87.5%, respectively [39]. Another study in France revealed that none of the isolates carried a *bla*_SHV_ gene, whereas the *bla*_TEM-1_ gene was detected in 2.2% (3/137) isolates [37]. On the other hand, a study in China claimed that 68.2% and 16.7% of *K. pneumoniae* strains harbored *bla*_SHV_ and *bla*_CTX-M_, respectively [34]; these genes normally have broad hosts but are often found in *E. coli* and *Klebsiella* spp. [40]. Sulphonamides are widely used due to their low costs and resistance, primarily mediated by the *sul1 *and *sul2* genes encoding dihydropteroate synthetase with a low affinity for sulphonamides [41]. Sulphonamide resistance in the current study varied depending on the antimicrobials (STX/SUX/SUL) used. A similar study in Pakistan reported that the proportion of *sul*1 was 39% and *sul*2 was 44% [42]; compared with this finding, our results were far lower, and resistance to sulphonamides may be less affected by these genes and need further investigation.

Resistance to tetracycline is governed by *tet* genes, which are involved in either the active efflux of the drug, ribosomal protection, or enzymatic drug modification. Among the various* tet* genes, *tet*A and *tet*B are reported in Gram-negative bacteria [43]. A similar study in China reported that 30.3% *K. pneumoniae* carried *tet*A gene [34], whereas a study in the Czech claimed that *tet*A prevalence of 5% and no *tet*B in the dairy farm [44]; compared to these results, our results were much higher in *tet*A and similar in *tet*B proportion. Although all isolates harbored *tet*A gene, the resistance to tetracycline in the current study was only 6%. It may be due to the other resistance mechanisms and need future studies. One of the three main mechanisms of quinolone resistance has been described as the acquisition of plasmid-mediated quinolone resistance genes leads to the protection of quinolone’s targets by *qnr* proteins [45]. A study in Egypt reported that out of fourteen *Klebsiella* spp. strains, two isolates carried *qnr*A gene, which accounted for 14.3% [46]. Connected to the resistant profile in the current study, the proportion of quinolone resistance was less than 4% and trimethoprim 2%, which may link to the lack of *qnr*A and DHFR-I genes. 

The severity of infections caused by *Klebsiella* spp. will depend on many factors, including the virulence profile of each isolate. The polysaccharide capsule is one of the most important virulence factors [47], which is encoded by *mag*A (mucoviscosity-associated gene A and specific to K1 capsule serotype), *k*2A (specific to K2 capsule serotype), and *rmp*A [20]. Additionally, siderophores which is the ability to procure iron for bacteria growth and survival, are encoded by the *ent*B, *iut*A, *iro*N genes [48]. Type 1 fimbriae are thread-like protrusions on the bacterial cell surface, and the tip adhesive subunit is encoded by *fim*H [49].

Similar studies in China reported that the prevalence of *fim*H was 100% isolates, *iro*N 4.4%, *rmp*A 4.4% [31]; *ent*B (78%), *fim*H1 (55%), *kfu* (31%), and *mrk*D (24%) were the prevalence of virulence genes among *K. pneumoniae*, whereas *ent*B (50%), *fim*H1 (30%), and *mrk*D (22%) were prevalent in *K. oxytoca *[10], none of the isolates found K1, K2 but 100% isolate carried *ent*B, 12% *iut*A, 94% *fim*H, and 3% *rmp*A [48]. Compared to these findings, our results look different in some virulence genes but similar in *fim*H and* ent*B, which may be the main virulence factors of *Klebsiella* spp. from dairy animals in Vietnam and China. Significant differences in the frequency of antibiotic-resistant and virulence genes in different studies may be due to different sources of isolation in different regions, their methods of investigation and sensitivity, and the number and types of samples.

## Conclusion

In conclusion, this is the first study to report the occurrence and molecular characterization of *Klebsiella *spp. from dairy animals, especially in Vietnam. Our study indicated that the infection of *Klebsiella *spp. associated with bovine mastitis are mostly multidrug-resistant, which increases the difficulties of treatment for mammary infections and is also a potential public health concern. Surprisingly, none of the isolates were ESBL producers, which was confirmed by the DDT test. This pathogen carried virulence genes, including adhesion (*fim*H), siderophore (*ent*B), and antimicrobials resistant genes (*bla*_SHV_, *acr*A_Kp_, *tet*A, etc.). Results from this study provided information about the distribution and characteristics of pathogenic *Klebsiella* spp.-infected bovine mastitis that could support dairy farmers/ owners and veterinarians to improve the usage and management of antimicrobial in mastitis treatment, especially with *Klebsiella *mastitis in large-scale herds.

## List of abbreviations

ATCC, American Type Culture Collection; CLSI, Clinical and Laboratory Standards Institute; DNA, Deoxyribonucleic acid; DDT, Disk diffusion test; ESBL, Extended-spectrum beta-lactamase; MDR, Multi-drug resistance; MHA, Mueller-Hinton agar; PCR, Polymerase chain reaction
